# Prevalence and Characteristics of* Salmonella* Isolated from Free-Range Chickens in Shandong Province, China

**DOI:** 10.1155/2016/8183931

**Published:** 2016-10-09

**Authors:** Xiaonan Zhao, Yanxia Gao, Chaoqun Ye, Lingling Yang, Tao Wang, Weishan Chang

**Affiliations:** ^1^College of Animal Science and Technology, Shandong Agricultural University, Tai'an 271000, China; ^2^College of Life Sciences, Taishan Medical University, Tai'an 271000, China

## Abstract

Compared with chickens raised in intensively managed breeding farms, free-range chickens in China are quite popular due to lower breeding density and less antibiotics usage. However, investigations about* Salmonella enterica* from free-range chickens are quite rare. The aim of the present study was to investigate prevalence and characteristics of* Salmonella* in free-range chickens in Shandong province, China. During the period of August and November 2015, 300 fresh fecal swabs from different broilers in three free-range chicken farms (100 samples per farm) were collected to isolate* Salmonella*, and then these isolates were subjected to serotyping, antibiotic sensitivity testing, enterobacterial repetitive intergenic consensus-polymerase chain reaction (ERIC-PCR), and multilocus sequence typing (ST). A total of 38* Salmonella* isolates (38/300, 12.7%) were recovered. The most common serotype was Enteritidis (81.6%), followed by Indiana (13.2%) and Typhimurium (5.3%). Twenty-two out of 38 isolates (57.9%) were resistant to ampicillin, the highest resistance rate, but resistance rates to cefazolin, cefotaxime, and ceftazidime were only 7.9%. The multidrug resistance (MDR) rate was 26.3%. Additionally, the* Salmonella* isolates could be classified into 25 genotypes by ERIC-PCR and were divided into three ST types (ST11, ST17, and ST19), with ST11 the highest isolation rate (81.6%). In summary, as with other poultry, free-ranging chickens may also serve as potential reservoir for antibiotic resistant* Salmonella*, thereby posing a threat to public health.

## 1. Introduction

*Salmonella enterica* is one of the most important pathogenic bacterial causes of food-borne diseases [1]. At present, more than 2,600 serotypes were identified, and most serotypes can cause food-borne infection [2].* Salmonella* infections can result in gastrointestinal problems such as gastroenteritis, typhoid fever, and paratyphoid fever or even cause death in serious infections, especially for younger people and the elderly [3–5]. In China, bacterial food-borne disease cases are frequently caused by* Salmonella*, and* Salmonella* in food-producing animals is a threat to public health [6, 7]. Food-producing animals especially chickens and pigs are regarded as the most important* Salmonella* carriers. Epidemiological investigations of* Salmonella* from chickens, pigs, and their meat products have shown that drug resistance of* Salmonella* has been increasing in China and other countries [7–10].

In recent years, free-range chicken farms are major contributors to organic food production in Shandong province, China. The free-range chicken farms are divided by spatially separated (chickens and crops are physically isolated), rotational (chickens can roam into field only after crops are harvested), and fully combined (chickens and crops are allowed to interact freely) farming. Importantly, although the chickens are purchased from local commercial poultry hatchery, fewer antibiotics are used for therapy or growth promotion. Compared with chickens raised in intensively managed breeding farms, free-range chickens in China are quite popular due to lower breeding density and less antibiotics usage.

However, investigations about* Salmonella* from free-ranging chickens are quite rare in China. Therefore, the aim of the present study was to isolate* Salmonella* from free-ranging chickens in Shandong province, China, and then these isolates were subjected to serotyping, antibiotic sensitivity testing, enterobacterial repetitive intergenic consensus-polymerase chain reaction (ERIC-PCR), and multilocus sequence typing (ST).

## 2. Materials and Methods

### 2.1. Sample Collection and Process

Between August and November 2015, 300 fresh fecal swabs of chickens aged about 6 weeks (100 samples per farm) were obtained from three free-range chicken farms (Jinan, Linyi, and Laiwu) (about 500 chickens per farm) in Shandong province, China. Fresh fecal samples were collected from near the chickens using sterile cotton swabs. After sampling, the swabs were transported to our lab in an ice box and processed within 6 h. The cotton swabs were immediately put into vials containing 100 mL of germ-free BPW media and shaken sufficiently at 37°C for 18 to 20 h. Then 0.5 mL and 0.1 mL of the enrichment broth were added to 10 mL of Tetrathionate Broth (TT, Becton-Dickinson, USA) and Rappaport-Vassiliadis Medium (RV, Becton-Dickinson, USA), respectively, at 42°C ± 1, 100 ppm for 22 to 24 h. Then, the bacteria in TT and RV were inoculated to xylose lysine tergitol 4 (XLT4, Becton-Dickinson, USA) agar and incubated at 37°C ± 1 overnight. Suspected* Salmonella* colonies were then inoculated to trisaccharide agar slant and incubated at 35°C ± 1 for 24 h. The typical* Salmonella* colonies were identified by the VITEK system (BioMerieux, Marcy l'Etoile, France) and then verified by PCR amplification of inherent gene* invA* [11].

### 2.2. Salmonella Serotyping

Commercial serodiagnosis kits for* Salmonella* (Ningbo Tianrun Bio-pharmaceutical Co., Ltd., China) were used to carry out the plate agglutination tests and identify serotypes of* Salmonella*. The serotyping scheme was referenced to the Kauffman-White salmonella serotyping scheme [12].

### 2.3. Antimicrobial Susceptibility Testing

A panel of 14 antibiotics, amikacin (AMK), gentamicin (GEN), kanamycin (K), norfloxacin (NOR), ampicillin (AMP), cefazolin (CFZ), cephradine (RAD), cefotaxime (CTX), chloramphenicol (CHL), tetracycline (TET), co-trimoxazole (SXT), ceftazidime (CAZ), ciprofloxacin (CIP), and doxycycline (DOX), was used to test the antimicrobial susceptibility of* Salmonella* by Kirby-Bauer disk diffusion method, as recommended by the Clinical and Laboratory Standards Institute [13]. An isolate was considered as multidrug-resistant (MDR) when exhibiting resistance to antimicrobials of at least three different classes [14].* Escherichia coli* ATCC 25922 and* Pseudomonas aeruginosa* ATCC 27853 were used as quality control strains.

### 2.4. ERIC-PCR

The primers ERIC 1 (5′-ATGTAAGCTCCTGGGATTCAC-3′) and ERIC 2 (5′-AAGTAAGTGACTGGGGTGAGCG-3′) were synthesized by Takara Biotechnology Co., Ltd. (Dalian, China). The total volume of PCR reaction was 25 *μ*L, including 10x buffer 2.5 *μ*L, 2.5 mmol/L dNTP 2.0 *μ*L, forward and reverse primers (20 pmol/L) 1 *μ*L for each, 25 mmol/L MgCl_2_ 1.5 *μ*L, Taq polymerase 5 U, and genome DNA 1.5 *μ*L. The ERIC-PCR was completed by an initial heat activation of 10 min at 94°C, then 31 cycles of 30 s at 92°C and 60 s at 40°C, 8 min at 65°C, and an extension of 8 min at 65°C. PCR products were run on an agarose gel electrophoresis (2%) at 100 V for 1 h [15].

### 2.5. ERIC-PCR Fingerprint Analysis

The samples that could be amplified by ERIC-PCR were marked as “1”; otherwise, they were marked as “0.” They were submitted to Gel Image System (Version 4.00) to give a matrix graph automatically. The unweighted pair group method using averages algorithm (UPGMA) was used to obtain the clustering dendrograms (NTSYS-pc 2.10 software). The individual isolates were regarded as an independent operational taxonomic unit (OUT), and isolates with a similarity over 90% were assumed to be of the same origin [15].

### 2.6. MLST

The seven pairs of housekeeping genes for* Salmonella* MLST assays from University College Cork (http://mlst.ucc.ie/), aroC, dnaN, hemD, hisD, pure, sucA, and thrA, were used as indexes for gene classification. Finally, these results were compared to the* S. enterica* MLST database (http://mlst.warwick.ac.uk/mlst/dbs/Senterica) for the ST types [16].

## 3. Results

### 3.1. Isolation of* Salmonella*

In the present study, 38* Salmonella* isolates were obtained from 300 samples from three free-range chicken farms, including 10 isolates (numbers 1–10) from Jinan (10/100, 10%), 13 (numbers 11–23) from Linyi (13/100, 13%), and 15 (numbers 24–38) from Laiwu (15/100, 15%).

### 3.2. Serotypes of* Salmonella*

The 38* Salmonella* isolates were classified into three serotypes according to the plate agglutination tests. The most common serotype was* S*.* enterica* serovar Enteritidis (*n* = 31, 81.6%), followed by* S*.* enterica* serovar Indiana (*n* = 5, 13.2%) and* S*.* enterica* serovar Typhimurium (*n* = 2, 5.3%) (Table 1).

### 3.3. Antimicrobial Susceptibility Testing

Drug resistance rates for the 38* Salmonella* isolates (Table 3) were as follows: ampicillin, 57.9% (22/38); co-trimoxazole and kanamycin, 29.0% each (11/38); tetracycline, 26.3% (10/38); doxycycline and gentamicin, 23.7% each (9/38); and cefazolin, cefotaxime, and ceftazidime, 7.9% each (3/38). Ten of thirty-eight isolates (26.3%) were MDR (Tables 2 and 3).

### 3.4. ERIC-PCR Analysis

**Figure 1:**
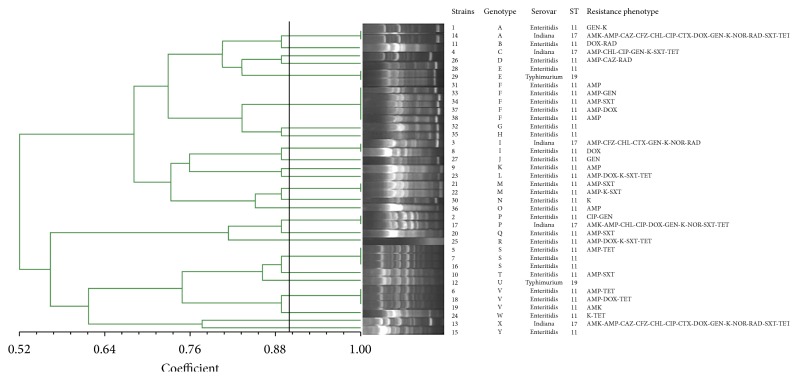
Dendrogram of* Salmonella* isolates from free-range chickens by ERIC-PCR.

The 38* Salmonella* isolates could be divided into 25 genotypes by ERIC-PCR. Genetic similarity ranged from 52% to 100%. Of note, the genetic similarity of the following isolates was 100%: numbers 1 and 14; numbers 2 and 17; numbers 3 and 8; numbers 21 and 22; numbers 28 and 29; numbers 5, 7, and 16; numbers 6, 18, and 19; and numbers 31, 33, 34, 37, and 38 (Figure 1).

### 3.5. MLST

The thirty-eight* Salmonella* isolates were classified into three ST types. ST11 was the highest isolate rate (31/38, 81.6%), which belongs to the clonal complex 258 (CC258), followed by ST17 (5/38, 13.2%) and ST19 (2/3, 85.3%) (Figure 1).

## 4. Discussion

*Salmonella* is an important pathogen of great importance in public health. Poultry are considered to be important carrier of* Salmonella* [17, 18]. Numerous studies have been conducted on* Salmonella* isolated from intensive breeding chicken farms, slaughter houses, and chicken meats [2, 19–21], but little information is available on free-range chickens. In the present study, the overall isolate rate of* Salmonella* in three free-range chickens was 12.7%, which was lower than that in intensive chicken farms in China (>35%) [22].

Numerous* Salmonella* serotypes are pathogenic, including* S*.* enterica* serovar Enteritidis and* S*.* enterica* serovar Typhimurium [23]. The most common serotype identified in the present study was* S*.* enterica* serovar Enteritidis (81.6%). It was consistent with investigation results from the intensively managed chicken farms in the Henan and Sichuan areas of China [24, 25]. But the most common isolated* Salmonella* from the intensively managed chicken farms in Cambodia, Vietnam, and South Korea were* S*.* enterica* serovar Anatum,* S*.* enterica* serovar Infantis, and* S*.* enterica* serovar Hadar, respectively [17, 26, 27]. The difference of the* Salmonella* serotype distribution may mainly be related with area differences.

Compared with the resistant rate of* Salmonella* from intensive poultry farms in Shandong province, China, in 2012, in the present study, the resistant rate of these 38* Salmonella* isolates against ampicillin, kanamycin, co-trimoxazole, and tetracycline was greatly lower (36.1–72.3% versus 26.3–57.9%) [28]. This apparent difference may be attributable to less drug use in free-range chickens. Of note,* Salmonella* isolates in this study were relatively sensitive to cephalosporin antibiotics (resistance rate, 7.9–13.2%), and these values are lower than the recorded values for cephalosporin antibiotics resistance in chickens raised in intensively managed farms in Shandong province, China (resistance rate, about 42% in 2012) [28]. Our findings showed that 26.3% of* Salmonella* isolates were MDR. This was lower than that in intensive poultry farms of Henan (46.0%) province in China [24]. Of note, in this study 2 out of 5* S*.* enterica* serovar Indiana isolates were resistant to 14 antibiotics, and they were not only resistant to streptomycin and tetracycline but also resistant to chloramphenicol, fluoroquinolones, and cephalosporin antibiotics. The antibiotic resistance difference of* Salmonella* isolated from three free-range chicken farms may be due to the fact that the use of antibiotics in different regions is quite different and the distribution of the antibiotics in the environments, such as waters and soils, has a great effect on drug resistance of* Salmonella*.

The result of ERIC-PCR showed that the 38* Salmonella* isolates were classified into 25 gene types, which indicates that the sources of* Salmonella* isolates in these flocks were diverse. Compared with* Salmonella* isolates from Laiwu, the isolates from Jinan and Linyi had lower genetic similarity. The difference should be further studied. The fact that isolates from Laiwu had higher genetic similarity may be associated with clonal spread of* Salmonella*. Thirty-eight* Salmonella* isolates were classified into 3 ST types (ST11, ST17, and ST19). All three types have also been isolated from human samples [29–31], suggesting that these* Salmonella* could be spread between human beings and chickens via food chain and threaten the human health.

## 5. Conclusions

Collectively,* Salmonella* isolated from free-ranging chickens showed relatively lower resistance rates than those raised in intensively managed chicken farms of China. These results also revealed that free-ranging chickens may serve as a potential reservoir for antibiotic resistance* Salmonella*, thereby posing a threat to public health.

## Figures and Tables

**Table 1:** Serotyping of *Salmonella* isolated from three free-range chicken farms.

Serovar	Number of isolates			Total (*n* = 38)
	Jinan (*n* = 10)	Linyi (*n* = 13)	Laiwu (*n* = 15)	
Enteritidis	8 (80%)	9 (69.2%)	14 (93.3%)	31 (81.6%)
Indiana	2 (20%)	3 (23.0%)	0	5 (13.2%)
Typhimurium	0	1 (7.7%)	1 (6.7%)	2 (5.3%)

**Table 2:** Antibiotic resistance rates of *Salmonella* isolated from three free-range chicken farms.

Antimicrobials	Antibiotics resistance rates *N* (%)
Amikacin	4 (10.5%)
Ampicillin	22 (57.9%)
Cefazolin	3 (7.9%)
Cefotaxime	3 (7.9%)
Ceftazidime	3 (7.9%)
Cephradine	5 (13.2%)
Chloramphenicol	5 (13.2%)
Ciprofloxacin	5 (13.2%)
Co-trimoxazole	11 (29.0%)
Doxycycline	9 (23.7%)
Gentamicin	9 (23.7%)
Kanamycin	11 (29.0%)
Norfloxacin	4 (10.5%)
Tetracycline	10 (26.3%)

**Table 3:** Antimicrobial resistance patterns of *Salmonella* isolated from three free-range chicken farms.

Resistant patterns	Number of resistant isolates			
	Enteritidis	Indiana	Typhimurium	Total
AMK-AMP-CAZ-CFZ-CHL-CIP-CTX-DOX-GEN-K-NOR-RAD-SXT-TET	0	2	0	2
AMK-AMP-CHL-CIP-DOX-GEN-NOR-K-SXT-TET	0	1	0	1
AMK-CFZ-CHL-CTX-GEN-K-NOR-RAD	0	1	0	1
AMP-CHL-CIP-GEN-K-SXT-TET	0	1	0	1
AMP-DOX-K-SXT-TET	2	0	0	2
AMP-CAZ-RAD	1	0	0	1
AMP-DOX-TET	1	0	0	1
AMP-K-SXT	1	0	0	1
AMP-DOX	1	0	0	1
AMP-GEN	1	0	0	1
AMP-SXT	4	0	0	4
AMP-TET	2	0	0	2
CIP-GEN	1	0	0	1
GEN-K	1	0	0	1
K-TET	1	0	0	1
DOX-RAD	1	0	0	1
AMK	1	0	0	1
AMP	4	0	0	4
GEN	1	0	0	1
DOX	1	0	0	1
K	1	0	0	1
